# Imaging of SARS-CoV-2 infected Vero E6 cells by helium ion microscopy

**DOI:** 10.3762/bjnano.12.13

**Published:** 2021-02-02

**Authors:** Natalie Frese, Patrick Schmerer, Martin Wortmann, Matthias Schürmann, Matthias König, Michael Westphal, Friedemann Weber, Holger Sudhoff, Armin Gölzhäuser

**Affiliations:** 1 Physics of Supramolecular Systems and Surfaces, Faculty of Physics, Bielefeld University, Bielefeld, Germany; 2 Institute of Virology, Faculty of Veterinary Medicine, Justus-Liebig-University Giessen, Germany; 3 Faculty of Engineering and Mathematics, Bielefeld University of Applied Sciences, Bielefeld, Germany; 4 University Clinic for Otolaryngology, Head and Neck Surgery, Medical Faculty OWL at Bielefeld University, Germany

**Keywords:** bioimaging, cell membrane, charge compensation, helium ion microscopy, SARS-CoV-2, Vero E6 cells

## Abstract

Helium ion microscopy (HIM) offers the opportunity to obtain direct views of biological samples such as cellular structures, virus particles, and microbial interactions. Imaging with the HIM combines sub-nanometer resolution, large depth of field, and high surface sensitivity. Due to its charge compensation capability, the HIM can image insulating biological samples without additional conductive coatings. Here, we present an exploratory HIM study of SARS-CoV-2 infected Vero E6 cells, in which several areas of interaction between cells and virus particles, as well as among virus particles, were imaged. The HIM pictures show the three-dimensional appearance of SARS-CoV-2 and the surface of Vero E6 cells at a multiplicity of infection of approximately 1 with great morphological detail. The absence of a conductive coating allows for a distinction between virus particles bound to the cell membrane and virus particles lying on top of the membrane. After prolonged imaging, it was found that ion-induced deposition of hydrocarbons from the vacuum renders the sample sufficiently conductive to allow for imaging even without charge compensation. The presented images demonstrate the potential of the HIM in bioimaging, especially for the imaging of interactions between viruses and their host organisms.

## Introduction

The last decade of helium ion microscopy (HIM) was characterized by a rapid exploration of its sub-nanometer imaging and ion-beam nanofabrication capabilities in materials science and engineering [1]. Although HIM soon proved to be a promising tool in the life sciences, the examination of biological samples by HIM proceeded at a much slower pace. In recent years, it has been used in the field of cell biology for imaging various human and animal cells. These include cartilage [2], cancer [3], liver [4], kidney [5] and stem cells [6], as well as fibrin fibers [7]. To visualize viruses and their host organisms, HIM has so far been applied to image T4 phage-infected *E. coli* bacteria [8], various phases of the life cycle of the bacterial predator *Bdellovibrio bacteriovorus* [9] and the vesicular structure of ethane-oxidizing archaea [10]. A comprehensive review on the subject of bioimaging with HIM has recently been published by Schmidt and co-workers [11].

In this work, we use HIM to investigate Vero E6 cells infected with the novel severe acute respiratory syndrome coronavirus 2 (SARS-CoV-2). Several members of the family *Coronaviridae* have been described in the human population and usually cause mild respiratory disease. SARS-CoV-2 demonstrated a world-wide spread causing a significant global public health emergency [12–13]. As of January 18th, 2021, more than 95 million cases worldwide have been confirmed with the infection and over two million infected patients have died [14]. African green monkey kidney Vero E6 cells have been reported to support SARS-CoV-2 replication in culture, while many more cell lines have been reported to be refractory to SARS-CoV-2 infection [15]. Both scanning electron microscopy (SEM) and transmission electron microscopy (TEM) have been used to image SARS-CoV-2 [16–20]. While TEM achieves unsurpassed resolution and can visualize macromolecular structures such as spike glycoproteins or transmembrane proteins [21], SEM provides topographic images of infected cells and virus particles distributed on their surface, albeit only after the samples have been coated with a conductive layer. In contrast, the HIM delivers a topographic image of the uncoated surface morphology of cells and virus particles, allowing one to identify and investigate sites at which a cell interacts with the virus. While its principle of operation is very similar to SEM, HIM utilizes a beam of positively charged helium ions (He^+^) instead of negatively charged electrons to excite and detect secondary electrons from the sample surface. Due to the high brightness and low energy spread of its atomically sharp gas field ion source, the smallest attainable focused spot size is about 0.3 nm [22]. With its significantly smaller convergence angle compared to SEM, HIM achieves a much larger depth of field, which is particularly useful for imaging three-dimensional structures [22]. Due to their higher mass, He^+^ ions penetrate deeper into the sample and do not spread as wide as electrons, resulting in a smaller escape volume of the secondary electrons and a higher surface resolution of the HIM, compared to the SEM [23]. A further benefit of HIM is its charge compensation capability during secondary electron detection. SEM imaging of biological specimen usually necessitates a thin conductive coating to prevent negative charge accumulation from the impinging electrons. Such coatings, albeit only a few nanometers thick, can significantly alter and conceal fine details of biological nanostructures [2], which is noticeable in SEM images of virus particles [19,24]. Since in the HIM positive charge accumulates on insulating samples, a low-energy electron flood gun can be used for charge compensation, which irradiates the sample with a diffuse beam of electrons. This eliminates the need for a conductive coating, and allows for a direct view on nanoscale structures [6,25]. Here, we demonstrate the benefits of high-resolution HIM by imaging SARS-CoV-2 interacting with Vero E6 cells without any conductive coating. The presented images allow for the identification of SARS-CoV-2 virus particles, their interaction with the cell membrane and a distinction between virus particles bound to the cell surface from those lying on it.

## Experimental

Vero E6 cells were cultivated in Dulbecco's modified Eagle's medium (Thermo Fisher Scientific) supplemented with 10% fetal bovine serum (Capricorn Scientific) in a 5% CO_2_ atmosphere at 37 °C. SARS-CoV-2 (strain SARS-CoV-2 /München-1.2/2020/984, p.2) [26] was grown on Vero E6 cells and titrated as described [27]. Infection experiments were done under biosafety level 3 conditions with enhanced respiratory personal protection equipment.

For HIM, cells were seeded onto coverslips placed in 24-well plates. The coverslips were previously sputter coated with 30 nm of gold to improve charge neutralization during HIM imaging. After 24 h, nearly confluent monolayers were infected with SARS-CoV-2 at a multiplicity of infection (MOI) of approximately 1 or mock-infected using cell culture medium. Following an incubation period of 18 h in a cell culture incubator (37 °C), cells were washed with 0.1 M sodium cacodylate (NaCac, pH 7.4) and fixed in 2% (v/v) glutaraldehyde, 2% (w/v) paraformaldehyde in NaCac buffer at room temperature for 30 min. After fixation at room temperature, the samples were transferred to the normal laboratory area and then fixed at 4 °C with fresh fixatives. The coverslips were subsequently washed and dehydrated in a graded series of ethanol (50%, 70%, 95%, 99.5% (2×)), transferred to water-free acetone and critical point dried in carbon dioxide.

HIM was performed with an Orion Plus microscope (Carl Zeiss) at an acceleration voltage of about 36 kV and a working distance of 20 mm. The spot control was set to 6 to obtain a beam current of 0.2 to 0.4 pA. To avoid charging effects during secondary electron detection, an electron flood gun was used after each line scan, if not stated otherwise, with a flood energy of 540 eV, flood time of 10 µs and a focus of 107 V. It should be mentioned that the flood gun parameters have to be optimized for each magnification level. All HIM images were recorded with 1024 × 1024 pixels. Before imaging, each sample was stored in the vacuum chamber of the microscope at 3.3 × 10^−7^ mbar for at least 24 h to remove most volatile organic contaminants.

## Results and Discussion

**Figure 1:**
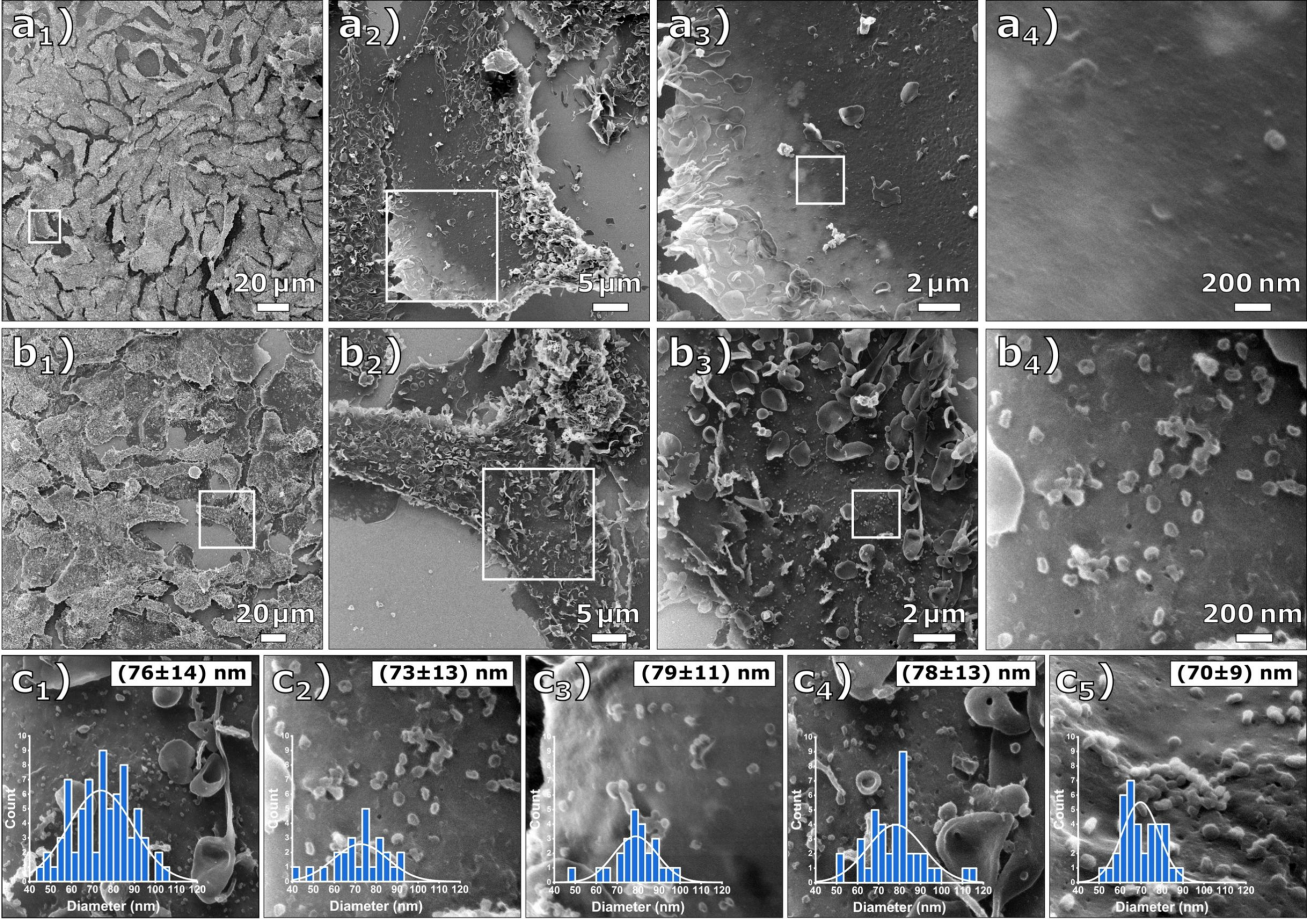
Comparative HIM images of Vero E6 cells that were mock-infected and infected at MOI 1. (a_1–4_) Mock-infected cells at different magnifications (FOV 200 µm, 45 µm, 15 µm, and 1.7 µm) and (b_1–4_) cells infected at MOI 1 at different magnifications (FOV 250 µm, 45 µm, 15 µm, and 1.7 µm). The cell membrane is covered with the virus particles. (c_1–5_) Determined virus particle diameter distributions. The inserted histograms show the respective image evaluation with normal distribution, mean value, and standard deviation. The average diameter of all evaluated images is 75 ± 13 nm.

A comparison between a native and an infected Vero E6 cell at multiple magnification levels is shown in Figure 1. Figure 1a shows a sequence of four HIM images of native Vero E6 cells (mock-infected). Figure 1b displays a sequence of HIM images of Vero E6 cells after they have been exposed to SARS-CoV-2 at a multiplicity of infection of approximately 1 (MOI 1) and an incubation time of 18 h. The surface of the infected cells is covered by a number of micrometer-sized vesicles and segments of cell membranes, which is a first indication that apoptosis occurred during viral replication. Regularly shaped particles below 100 nm diameter on the cell membrane shown in Figure 1b_4_ were only abundant on the cells of the MOI 1 sample and were therefore identified as SARS-CoV-2 virus particles. This is in accordance with a study of Bojkova et al. [28], which demonstrated the presence of newly synthesized viral particles of SARS-CoV-2 even 10 h after initial infection. The cell membrane of the infected cell is covered with the virus particles, which are predominantly spherically shaped. Holes in the cell membrane, illustrated in Figure 1b_4_ and Figure S1 of Supporting Information File 1, have previously been observed in uncoated mammalian cells and indicate lipid nanodomains or caveolea [6]. Figure 1c shows an evaluation of the virus particle size in five arbitrarily chosen regions on the MOI 1 sample resulting in an average diameter of the virus particles of 75 ± 13 nm, noting that this value has been obtained from viruses after fixation and critical point drying.

As He^+^ ions can penetrate several hundred nanometers into the sample [29], the outer rim of the cells appears brighter because the ions pass through the cells and generate additional secondary electrons at the back of the cells and in the gold-coated specimen slide [30]. The edges appear brightest where the cells bend upwards from the substrate. The edge resolution in two highly magnified images, shown in Figure S2 of Supporting Information File 1, has been determined by plotting the corresponding gray-scale values over the edges of two holes, resulting in values of 1.3 and 2.1 nm. The edge resolution of the images is determined by an interplay between the size of the focused He^+^ beam and the widening of the beam within the sample material. The obtained values are typical for biological materials [6–8,11].

**Figure 2:**
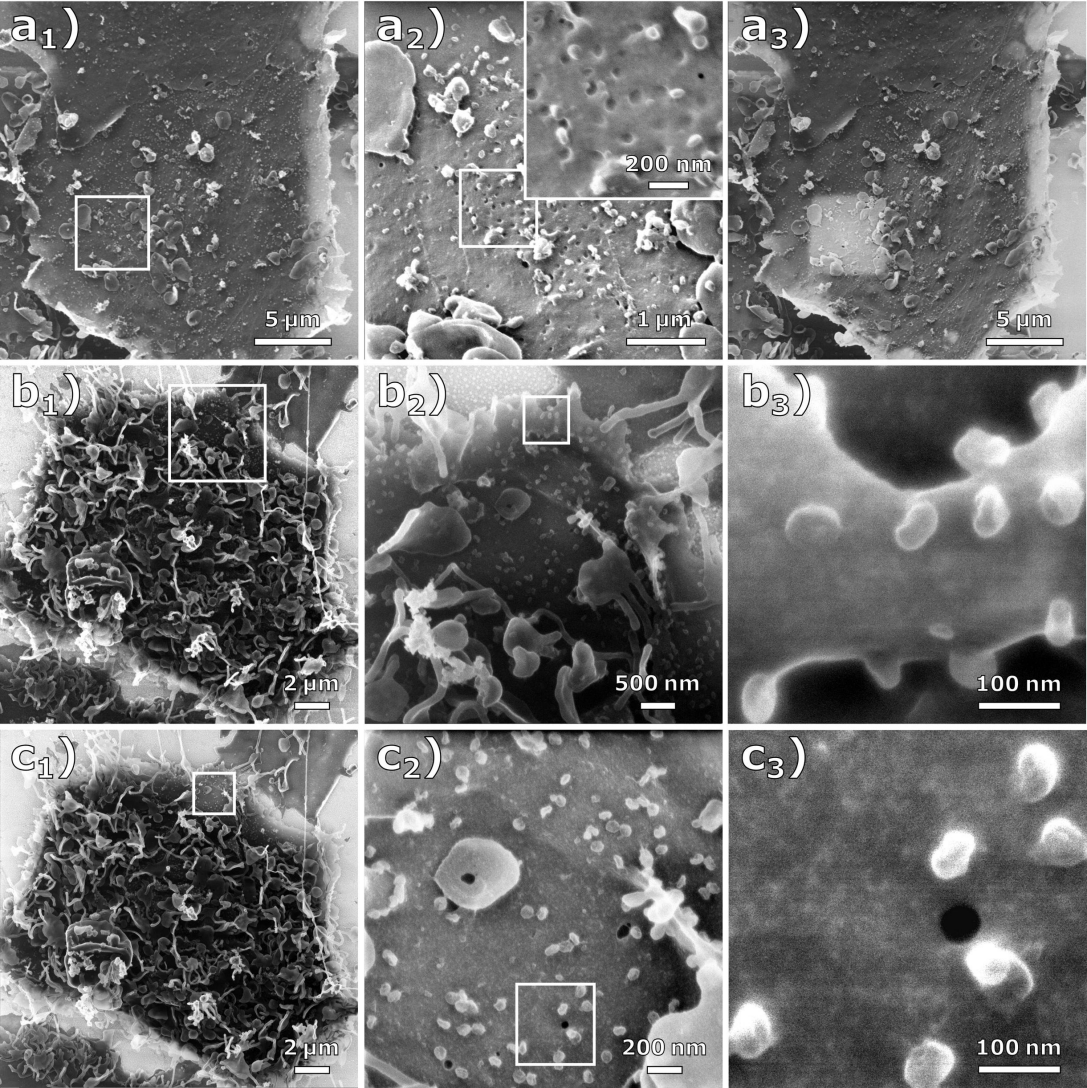
Effect of carbon deposition during HIM imaging. (a_1_) HIM image (FOV 20 µm) of a cell infected at MOI 1 with charge compensation. (a_2_) HIM images at high magnification (FOV 4.5 µm and 1 µm) with charge compensation. (a_3_) The same image section as (a_1_) after imaging the regions in (a_2_). Due to increased conductivity, this region appears significantly brighter than the rest of the image. (b_1–3_) HIM images of a cell infected at MOI 1 at different magnifications (FOV 20 µm, 5 µm, and 450 nm) with charge compensation. (c_1–3_) HIM images of the same cell (FOV 20 µm, 2 µm, and 450 nm) after imaging the magnified sections in (b). (c_1_) and (c_2_) were imaged with and (c_3_) was imaged without charge compensation.

An effect frequently occurring during HIM imaging with charge compensation can be observed in the sequence of HIM images shown in Figure 2a_1–3_, where a location on a MOI 1-infected Vero E6 sample was first imaged at a field of view (FOV) of 23 µm (Figure 2a_1_), followed by two higher magnification images with a FOV of 4.5 µm and a FOV of 1 µm (Figure 2a_2_). Figure 2a_3_ shows the same region as Figure 2a_1_, but the parts that were previously imaged at high magnification (FOV of 4.5 μm) with a dose of 1.4 × 10^16^ ions/cm^2^ appear noticeably brighter. This is caused by He^+^ beam-induced carbonaceous deposits resulting in a thin conductive coating. In addition to the improved conductivity of the specimen, the deposited layer may contribute to the electron density of the surface, thus increasing secondary electron yield. This effect, commonly referred to as electron- and/or ion beam-induced deposition, is commonly observed in charged-particle microscopes. In electron microscopes, deposition rates of up to 3 Å/s at high current densities have been reported. As the deposition rate quickly reaches an equilibrium with rising current density, it can be assumed that the limiting factor is the density of residual hydrocarbons in the vacuum [31]. In the HIM, residual gas as well as the specimen itself are considered the main contributors of hydrocarbons [32–33]. Due to the much larger mass of He^+^ ions compared to electrons, their sputter rate is typically much higher. Since organic compounds are ablated from the sample surface, hydrocarbon deposition is likely to be more pronounced when imaging biological samples in HIM. A schematic illustration of this effect can be seen in Figure S3 of Supporting Information File 1.

Figure 2b_1–3_ shows an infected Vero E6 cell at different magnification levels. Figure 2b_3_ depicts the highest magnification (FOV 450 nm) of the cell seen in Figure 2b_1_, showing the virus particles on top of the cell membrane in a side view. Note that after the zoom-out in Figure 2c_1_, the previously imaged regions appear again brighter. After imaging Figure 2c_2_ with a dose of 1.9 × 10^17^ ions/cm^2^, the flood gun was turned off, which allowed imaging of Figure 2c_3_ without any external charge compensation. From the quality of this image, it can be inferred that the deposited carbon layer rendered the sample sufficiently conductive. However, small structures are still visible on the membrane surface, which may originate from surface topography or material contrast. The deposited carbon film is presumably thinner than typical conductive metal or carbon coatings for SEM imaging, and it does not show any surface masking and clustering as seen on the gold substrate in the upper left of Figure 2b_2_. The energy of the incident hydrocarbons is much lower compared to the energy of sputter-deposited metals. However, it is possible that this unintended, but sometimes useful, carbon deposition can be reduced by HIM imaging in ultra-high vacuum [34–36].

**Figure 3:**
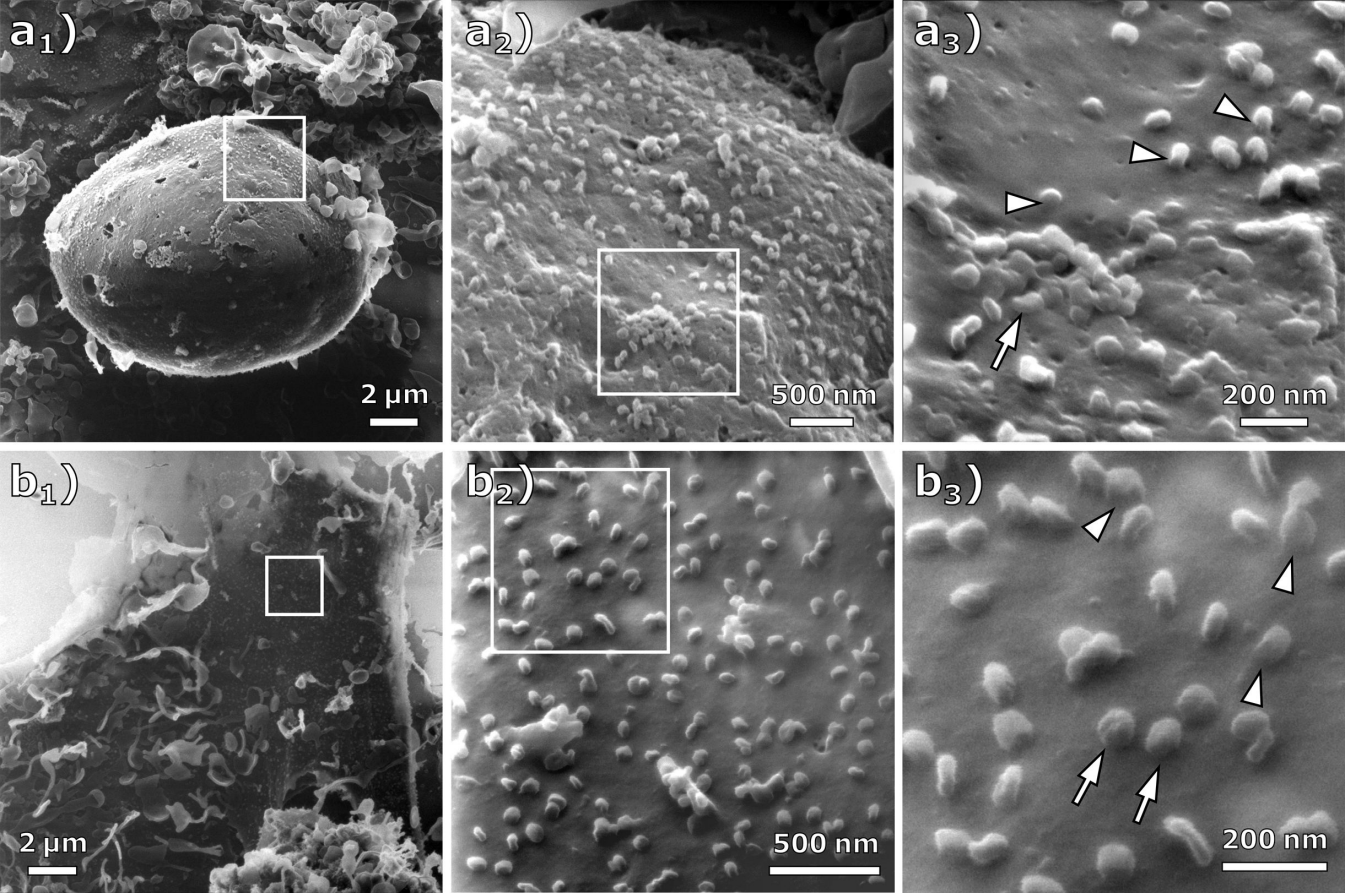
HIM images of cells infected at MOI 1 imaged with charge compensation. (a_1–3_) Different magnifications of an infected cell (FOV 17 µm, 3.5 µm, and 1.3 µm). At the high magnification in (a_3_), clusters of virus particles (arrow) and junctions (arrowheads) between the virus particle and the cell membrane become visible. (b_1–3_) Different magnifications of an infected cell (FOV 18 µm, 2 µm, and 850 nm). While some of the virus particles appear to be bound to the cell membrane (arrowheads), others seem to just lie on top of it (arrow).

The cell structures shown in the HIM images of Figure 3a are sharply resolved over tens of micrometers, which demonstrates the high depth of field of HIM compared to SEM [37]. In image 3a_3_, at the surface of the cell, a cluster of virus particles seems to be bound to the cell membrane (arrow). We suggest that this resembles the particle clustering by host defense protein BST-2 as it was observed for human coronavirus229E and quantified in HeLa cells by Wang and co-workers [38]. However, the metal coating applied by Wang et al. is clearly visible at high resolution in the SEM images as a rough layer on the cell membrane that hides the true topography [25,39]. In contrast, the HIM images presented here not only allow for the quantification of particles and clusters, but also enable an unveiled view on the interaction of virus particles with the cell membrane. The presented particle cluster seems to have a coalesced appearance, which might be caused by the virus–virus and virus–membrane interactions mediated through agglutinating BST-2 [40–41]. Some viral particles appear to be connected to the cell membrane by a continuous junction (arrowheads). Figure 3b shows another cell on the MOI 1 sample at different magnification levels. At the highest magnification shown in Figure 3b_3_ (FOV 850 nm), these junctions can also be observed (arrowheads). We assume that this resembles the tubulating cell membrane, which is stabilized by BST-2 to prevent viral scission. This alternative BST-2 interaction was already described for HIV-infected cells via immuno-TEM [42] but has not yet been observed for SARS-CoV-2. Aside from this observation, the HIM images allow for the distinction between viruses bound to the membrane and virus particles lying on top of the membrane (Figure 3b, arrows). Compared to a SEM study in which all visible virus particles on a cell membrane were quantified [38], HIM images could provide additional information about bound and unbound particles, resulting in more accurate data by counting only the bound particles. The presented images demonstrate that the HIM is well suited for the imaging of virus–membrane and virus–virus interactions, for example, when the virus particles are bound to the cell membrane or/and have a coalesced appearance.

It is known that the spike glycoproteins can be visualized by TEM. As the HIM images depicted the virus particles without conductive coating, it is an interesting question, whether or not the spike glycoproteins could, in principle, be resolved in HIM images. Inspecting the highest magnification images, Figure 2b_3_ and Figure 2c_3_, we do not see unequivocal evidence of structures indicating the spike glycoproteins. However, it is conceivable that a dedicated sample preparation could preserve their structure for imaging in HIM.

## Conclusion

In this study, HIM images of Vero E6 cells without infection and infected with SARS-CoV-2 are presented. On infected cells, the ultrastructure of the cell–virus interaction, as well as interaction among virus particles, is shown. The absence of a previously applied conductive coating allows for the distinction between virus particles bound to the cell membrane and virus particles lying on top of the cell membrane. The images unveil the three-dimensional appearance of SARS-COV-2 and the surface of Vero E6 cells at MOI 1 with an edge resolution of up to 1.3 nm. Additionally, it is shown that ion-induced deposition renders the sample surface sufficiently conductive to be imaged without charge compensation. The presented images demonstrate the potential of the HIM in bioimaging, especially for the imaging of interactions between viruses and their host organisms. HIM thus represents a versatile complement to conventional methods in the life sciences.

## Supporting Information

File 1 Additional experimental data.

## References

[1] Hlawacek G, Gölzhäuser A (2016). Helium Ion Microscopy.

[2] Vanden Berg-Foels W S, Scipioni L, Huynh C, Wen X (2012). J Microsc (Oxford, U K).

[3] BAZOU D, BEHAN G, REID C, BOLAND J J, ZHANG H Z (2011). J Microsc (Oxford, U K).

[4] Chen X, Udalagama C N B, Chen C-B, Bettiol A A, Pickard D S, Venkatesan T, Watt F (2011). Biophys J.

[5] Rice W L, Van Hoek A N, Păunescu T G, Huynh C, Goetze B, Singh B, Scipioni L, Stern L A, Brown D (2013). PLoS One.

[6] Schürmann M, Frese N, Beyer A, Heimann P, Widera D, Mönkemöller V, Huser T, Kaltschmidt B, Kaltschmidt C, Gölzhäuser A (2015). Small.

[7] Greiner J F W, Hauser S, Widera D, Müller J, Qunneis F, Zander C, Martin I, Mallah J, Schuetzmann D, Prante C (2011). Eur Cells Mater.

[8] Leppänen M, Sundberg L-R, Laanto E, de Freitas Almeida G M, Papponen P, Maasilta I J (2017). Adv Biosyst.

[9] Said N, Chatzinotas A, Schmidt M (2019). Adv Biosyst.

[10] Chen S-C, Musat N, Lechtenfeld O J, Paschke H, Schmidt M, Said N, Popp D, Calabrese F, Stryhanyuk H, Jaekel U (2019). Nature.

[11] Schmidt M, Byrne J M, Maasilta I J (2021). Beilstein J Nanotechnol.

[12] Corman V M, Lienau J, Witzenrath M (2019). Internist.

[13] Wu F, Zhao S, Yu B, Chen Y-M, Wang W, Song Z-G, Hu Y, Tao Z-W, Tian J-H, Pei Y-Y (2020). Nature.

[14] (2021). Coronavirus COVID-19 Dashboard, Johns Hopkins University.

[15] Takayama K (2020). Trends Pharmacol Sci.

[16] Zhu N, Zhang D, Wang W, Li X, Yang B, Song J, Zhao X, Huang B, Shi W, Lu R (2020). N Engl J Med.

[17] Algarroba G N, Rekawek P, Vahanian S A, Khullar P, Palaia T, Peltier M R, Chavez M R, Vintzileos A M (2020). Am J Obstet Gynecol.

[18] Harcourt J, Tamin A, Lu X, Kamili S, Sakthivel S K, Murray J, Queen K, Tao Y, Paden C R, Zhang J (2020). Emerging Infect Dis.

[19] Bouhaddou M, Memon D, Meyer B, White K M, Rezelj V V, Correa Marrero M, Polacco B J, Melnyk J E, Ulferts S, Kaake R M (2020). Cell.

[20] Prasad S, Potdar V, Cherian S, Abraham P, Basu A, ICMR-NIV NIC Team (2020). Indian J Med Res.

[21] Wolff G, Limpens R W A L, Zevenhoven-Dobbe J C, Laugks U, Zheng S, de Jong A W M, Koning R I, Agard D A, Grünewald K, Koster A J (2020). Science.

[22] Ward B W, Notte J A, Economou N P (2006). J Vac Sci Technol, B: Microelectron Nanometer Struct–Process, Meas, Phenom.

[23] Hlawacek G, Veligura V, van Gastel R, Poelsema B (2014). J Vac Sci Technol, B: Nanotechnol Microelectron: Mater, Process, Meas, Phenom.

[24] (2020). “New Images of Novel Coronavirus SARS-CoV-2 Now Available”, NIAID Media Team, Feb 17, 2020, USA.

[25] Joens M S, Huynh C, Kasuboski J M, Ferranti D, Sigal Y J, Zeitvogel F, Obst M, Burkhardt C J, Curran K P, Chalasani S H (2013). Sci Rep.

[26] Rothe C, Schunk M, Sothmann P, Bretzel G, Froeschl G, Wallrauch C, Zimmer T, Thiel V, Janke C, Guggemos W (2020). N Engl J Med.

[27] Felgenhauer U, Schoen A, Gad H H, Hartmann R, Schaubmar A R, Failing K, Drosten C, Weber F (2020). J Biol Chem.

[28] Bojkova D, Klann K, Koch B, Widera M, Krause D, Ciesek S, Cinatl J, Münch C (2020). Nature.

[29] Cohen-Tanugi D, Yao N (2008). J Appl Phys.

[30] Bell D C (2009). Microsc Microanal.

[31] Ennos A E (1953). Br J Appl Phys.

[32] Isaacson M (1979). Ultramicroscopy.

[33] Hren J J (1978). Ultramicroscopy.

[34] van Gastel R, Barriss L, Sanford C, Hlawacek G, Scipioni L, Merkle A P, Voci D, Fenner C, Zandvliet H J W, Poelsema B (2011). Microsc Microanal.

[35] Hlawacek G, Veligura V, Lorbek S, Mocking T F, George A, van Gastel R, Zandvliet H J W, Poelsema B (2012). Beilstein J Nanotechnol.

[36] Veligura V, Hlawacek G, van Gastel R, Zandvliet H J W, Poelsema B (2012). Beilstein J Nanotechnol.

[37] Wirtz T, De Castro O, Audinot J-N, Philipp P (2019). Annu Rev Anal Chem.

[38] Wang S-M, Huang K-J, Wang C-T (2014). Virology.

[39] Caldas L A, Carneiro F A, Higa L M, Monteiro F L, da Silva G P, da Costa L J, Durigon E L, Tanuri A, de Souza W (2020). Sci Rep.

[40] Mahauad‐Fernandez W D, Okeoma C M (2016). Immun, Inflammation Dis.

[41] Berry K N, Kober D L, Su A, Brett T J (2018). BioEssays.

[42] Hammonds J, Wang J-J, Yi H, Spearman P (2010). PLoS Pathog.

