# Supplementary material for: Imaging of SARS-CoV-2 infected Vero E6 cells by helium ion microscopy
Source: Beilstein J Nanotechnol. 2021 Feb 2;12:172–9. doi: 10.3762/bjnano.12.13 (PMC7871036; doi:10.3762/bjnano.12.13)
Supplement: File 1 — Additional experimental data. [file Beilstein_J_Nanotechnol-12-172-s001.pdf]

BEILSTEIN JOURNAL OF NANOTECHNOLOGY

Supporting Information

for

Imaging of SARS-CoV-2 infected Vero E6 cells by helium ion microscopy

Natalie Frese, Patrick Schmerer, Martin Wortmann, Matthias Schürmann, Matthias König, Michael Westphal, Friedemann Weber, Holger Sudhoff and Armin Gölzhäuser

Beilstein J. Nanotechnol. **2021,** *12,* 172–179. doi:10.3762/bjnano.12.13

Additional experimental data

License and Terms: This is a supporting information file under the terms of the Creative Commons Attribution License (https://creativecommons.org/licenses/by/4.0). Please note that the reuse, redistribution and reproduction in particular requires that the author(s) and source are credited and that individual graphics may be subject to special legal provisions.
The license is subject to the *Beilstein Journal of Nanotechnology* terms and conditions: (https://www.beilstein-journals.org/bjnano/terms)

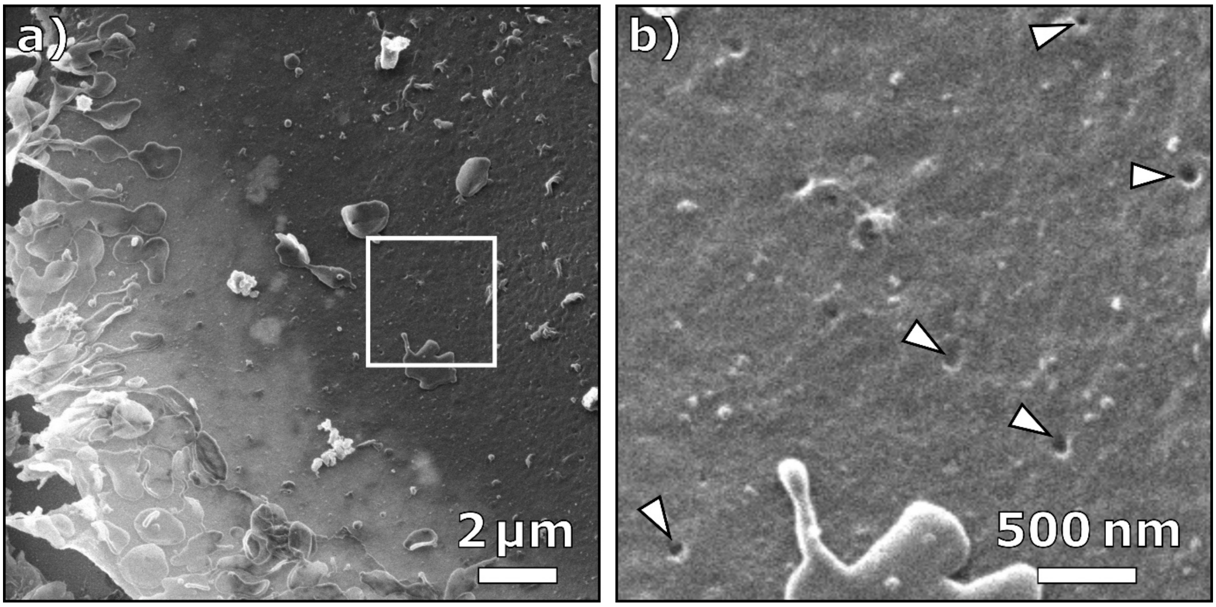

Figure S1: HIM images showing holes in the cell membrane (arrowheads) of a mock infected Vero E6 cell.

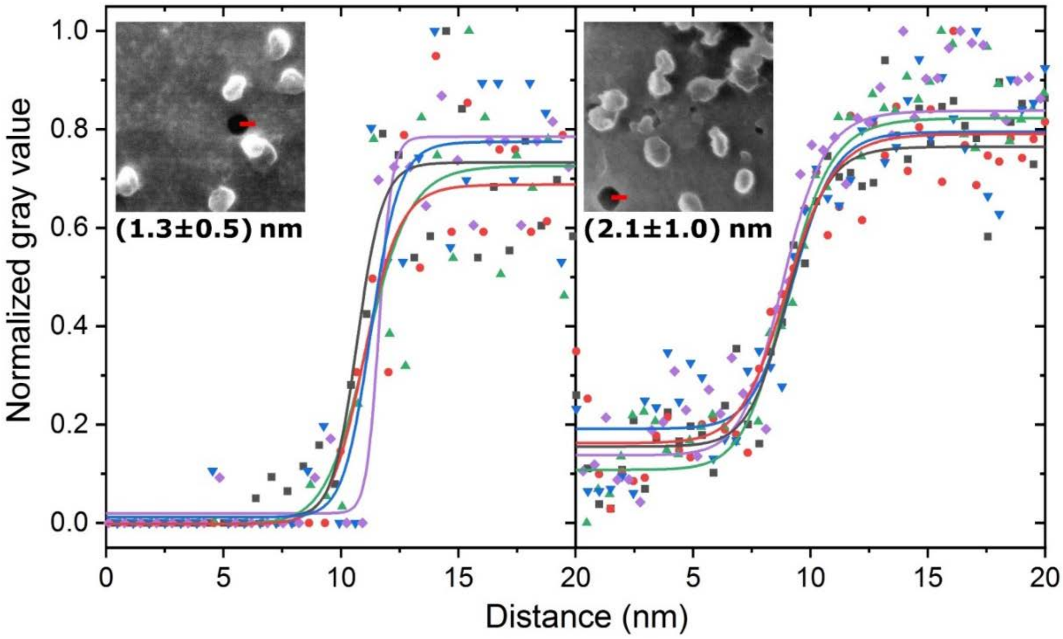

Figure S2: Edge resolution of two HIM images, averaged over five gray-scale plots each that were fitted by logistic regression. The corresponding edges of holes in the cell membrane are shown in the inserted images (red lines); respective mean values and standard deviations of $d_{25\%–75\%}$ are given below.

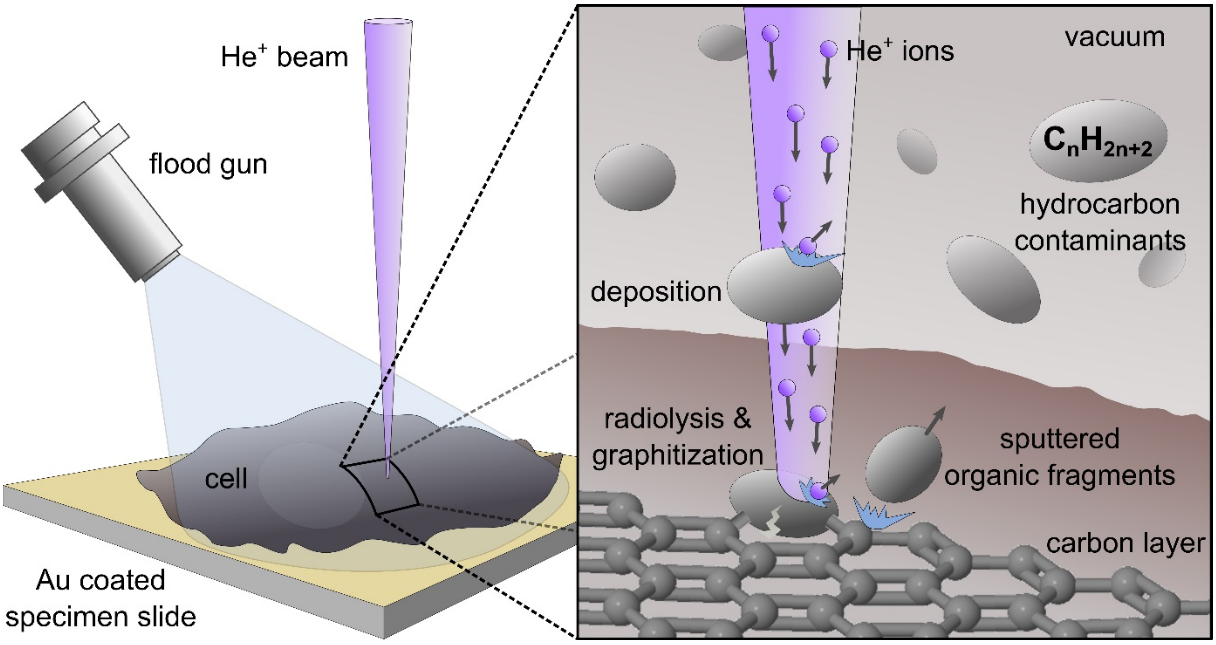

Figure S3: Schematic illustration of ion beam-induced deposition of hydrocarbons.
